# In vivo femtosecond laser nanosurgery of the cell wall enabling patch-clamp measurements on filamentous fungi

**DOI:** 10.1038/s41378-024-00664-x

**Published:** 2024-04-07

**Authors:** Tanja Pajić, Katarina Stevanović, Nataša V. Todorović, Aleksandar J. Krmpot, Miroslav Živić, Svetlana Savić-Šević, Steva M. Lević, Marina Stanić, Dejan Pantelić, Brana Jelenković, Mihailo D. Rabasović

**Affiliations:** 1 Institute of Physiology and Biochemistry “Ivan Djaja”, Faculty of Biology, University of Belgrade, Studentski trg 16, 11158 Belgrade, Serbia; 2 Institute for Biological Research “Siniša Stanković”, University of Belgrade, National Institute of the Republic of Serbia, Bulevar Despota Stefana 142, 11000 Belgrade, Serbia; 3 Institute of Physics Belgrade, University of Belgrade, National Institute of the Republic of Serbia, Pregrevica 118, 11080 Belgrade, Serbia; 4 University of Belgrade, Faculty of Agriculture, Nemanjina Street 6, 11080 Belgrade, Serbia; 5 Institute for Multidisciplinary Research, University of Belgrade, Kneza Višeslava 1, 11030 Belgrade, Serbia

**Keywords:** Optics and photonics, Nanofabrication and nanopatterning

## Abstract

Studying the membrane physiology of filamentous fungi is key to understanding their interactions with the environment and crucial for developing new therapeutic strategies for disease-causing pathogens. However, their plasma membrane has been inaccessible for a micron-sized patch-clamp pipette for pA current recordings due to the rigid chitinous cell wall. Here, we report the first femtosecond IR laser nanosurgery of the cell wall of the filamentous fungi, which enabled patch-clamp measurements on protoplasts released from hyphae. A reproducible and highly precise (diffraction-limited, submicron resolution) method for obtaining viable released protoplasts was developed. Protoplast release from the nanosurgery-generated incisions in the cell wall was achieved from different regions of the hyphae. The plasma membrane of the obtained protoplasts formed tight and high-resistance (GΩ) contacts with the recording pipette. The entire nanosurgical procedure followed by the patch-clamp technique could be completed in less than 1 hour. Compared to previous studies using heterologously expressed channels, this technique provides the opportunity to identify new ionic currents and to study the properties of the ion channels in the protoplasts of filamentous fungi in their native environment.

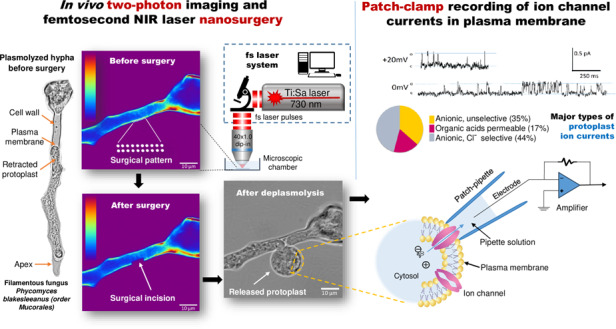

## Introduction

Filamentous fungi are an extremely diverse cosmopolitan group with great importance to the functioning of the biosphere. Together with bacteria, fungi represent the most important group of decomposers in almost all ecosystems, and mycorrhizal fungi form symbiotic associations with nearly 80% of land plants^1^. Moreover, the biodiversity of mycorrhizal fungi in soil has a critical impact on maintaining plant and ecosystem biodiversity^2^. In addition, some fungi are pathogenic and cause diseases in animals or plants^3^, and fungi that threaten human health have enormous biomedical importance^4^. Ion channels are known or expected to play important roles in fungal physiology^5^, including ion and nutrient uptake^6^, signal transduction^7^, and response to osmotic stress^8^. In contrast to animal and plant cells, little is known about the function of the ion channels in fungi. To date, only a handful of channels in filamentous fungi have been cloned and/or characterized by electrophysiological techniques; this has been performed mainly by heterologous expression of the channel proteins identified by screening for homologs of known yeast, animal or plant proteins^9–19^. The main reason for the near-complete lack of studies on native membranes is the rigid, chitinous cell wall that blocks access to the patch-clamp pipette. The cell wall makes it impossible to use the patch-clamp method; the gold standard for ion current measurements. For the membrane to be accessible by the glass pipette for the patch-clamp method, all or part of the wall must be removed. The naked protoplast (cellular cytoplasm whose entire contents are enclosed by the plasma membrane) would then be released through the opening in the hyphal wall to enable access with the patch-clamp pipette. The formation of a high-resistance contact (“seal”) between the tip of the glass pipette and the membrane is a prerequisite for the patch-clamp technique and is only possible if the membrane is clean^20^.

There are several ways to remove the cell wall: mechanically, enzymatically and by laser cell surgery^21,22^. Mechanical dissection of tissue to release a small number of protoplasts has never been successful in filamentous fungi, while this method is used in plant tissues to avoid the deleterious effects of enzymatic treatment on the plasma membrane^23^. Enzymatic removal of the cell wall was successful in plants^24,25^ but did not produce positive results in the filamentous fungi *Neurospora*^26^ and *Saprolegnia*^27^. Contacts with resistances greater than 200 and 500 MΩ were not possible to attain. These results were in contrast to plant findings and occurred because the fungal cell wall is structurally and molecularly distinct from the cellulose-based plant cell wall.

However, a small localized portion of the fungal cell, such as a tiny cell wall section, is possible to remove using the laser ablation technique^28^ while leaving the bulk of the wall intact. In this way, the polarity of the fungal cells is preserved, and a portion of the plasma membrane is accessible for patch-clamp measurements. Laser removal of the cell wall combined with patch-clamp recording has previously been used in some algae and plants^21,29–32^ and only in two filamentous fungi^10,33^. However, these experiments were conducted more than twenty years ago with UV lasers and did not evolve into a routine protocol. The UV lasers used in these studies operated with nanosecond (ns) laser pulses, which are known to cause photothermal and photomechanical damage to samples^34–36^. Recent developments in laser technology and microscopy, especially ultrafast femtosecond (fs) lasers, enable operations with extremely high precision. The main advantage of fs lasers is that they produce negligible thermal effects compared to ns lasers because their pulses are shorter than the thermal diffusion time (picoseconds to nanoseconds)^37^. Due to the nonthermal nature of the fs laser interacting with the cellular material, the material is mainly ablated^28,34,35,38^, causing minimal damage to the surrounding cellular material. In addition, the pulse energy for fs laser ablation is in the sub-nJ range, which is well below the pulse energies of UV-ns lasers. In addition, the wavelengths of fs lasers used for cell surgery are in the NIR range, which is not as harmful to the cell as UV wavelengths are; this aspect is particularly important for stray radiation during surgery.

Femtosecond lasers emit ultrashort laser pulses that allow a significant reduction in dissection size compared to ns lasers^34,35,38^. Thus, they are ideal for precise cell surgery at the nanoscale to microscale. In fs laser cell nanosurgery, a laser beam is focused down to the diffraction-limited focal spot/volume by a high numerical aperture (NA) objective lens on the cell wall or membrane^28,35,38^. In addition, the surgical process achieved with fs lasers enables the removal of very small amounts of material with high precision, creating a small incision on the cell wall/membrane or other cell structures. The high peak intensities of the fs laser pulses induce multiphoton interaction processes and provide a lower energy threshold for cell wall/membrane removal^28,35,38^. The typical peak intensity for fs laser ablation is on the order of 10^12^ W/cm^2^ ^39,40^. Briefly, ultrashort laser pulses with relatively low energy enable more precise submicrometer surgical incisions than ns lasers^34,35,38,41^.

Laser nanosurgery has become an important tool in many biological fields because of its precision, noninvasiveness, and versatility. This technique has been used as a microdissection tool for studying the function of microtubules, mitochondria and other organelles in cultured cells, tissues, and whole organisms, as well as for optotransfection and other forms of laser-based molecular delivery^42^. Other potential applications of this technology include developmental biology studies of cell mechanics, intracellular transport, and cell signaling, as well as the development of new therapies for diseases such as cancer and genetic disorders. In the context of fungi, fs laser nanosurgery could be used to selectively remove portions of the cell wall. The combination of laser ablation and localized patch clamping allows the measurement of ionic currents through the plasma membrane while retaining information about the location of the membrane from which recordings were made. This approach enables the detection of the asymmetric distribution of the ion channels along hyphae and the exploration of the ion channel involvement in the fungal cell polarity establishment, signal integration, and hyphal tropism; all of these play key roles in establishing fungal virulence^43^ and interactions with surrounding organisms or the environment^44,45^. Thus, we were motivated to develop and optimize a protocol that allows high-quality recording of currents via the patch-clamp method on the plasma membrane of fungal protoplasts released from the wall by subcellular nanosurgery using a fs titanium-sapphire (Ti:Sa) laser. Therefore, to the best of our knowledge, we present the first successful application of fs laser nanosurgery to access the plasma membrane of filamentous fungal hyphae for patch-clamp recording. We demonstrated that the released protoplasts could successfully form high-resistance contacts with a patch-clamp pipette. The incisions produced by the fs laser were smooth-edged, protoplasts were released from those incisions, and the probability of protoplast release was a function of the incision size and was modified by the presence of a high Ca^2+^ concentration. The method could be readily used for the selective release and subsequent recording of protoplasts from different regions (tip, middle, and side branches) of the fungal hyphae. Finally, we provided the first electrophysiological snapshot obtained on filamentous fungal protoplasts after cell wall removal by fs laser nanosurgery and showed that the protoplast membrane was characterized by a number of different types of ionic currents, predominantly anionic.

## Results

### In vivo two-photon imaging, cell wall laser nanosurgery and patch-clamp recording

**Fig. 1:**
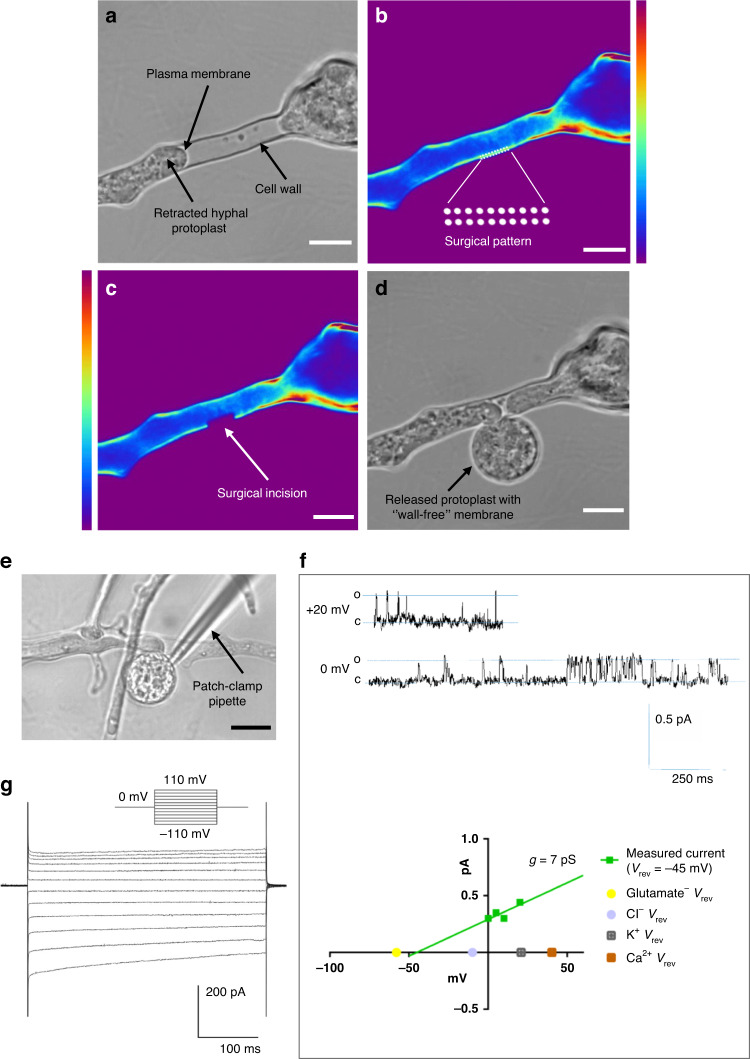
In vivo laser nanosurgery of the cell wall of the filamentous fungus *Phycomyces blakesleeanus* using fs laser pulses and patch-clamp recording of the released fungal protoplast membrane current activity after the surgical procedure. **a** Bright-field and **b** TPEF image of the plasmolyzed and labeled hypha before surgery. A 20×2 spot-wise pattern was positioned on the cytoplasm-free section of the cell wall. The average laser power in the sample plane for imaging was 1.1 mW (dwell time 2.5 µs) at 730 nm. **c** TPEF image of the same hypha after surgery. The surgical incision is indicated by the arrow. The laser power at the sample plane for the surgery was 6.1 mW (dwell time 1 s) at 730 nm. The color intensity bars for the TPEF signal are as follows: violet/blue, lowest TPEF signal; and dark red, highest TPEF signal. The color intensity bar is linear and covers the entire range of the data. Scale bar: 10 μm. **d** Bright-field image of the same hypha with the protoplast released through the surgical incision after laser cutting. **e** Bright-field image of the patch-clamp pipette in contact with the membrane of the protoplast released through the surgical incision. Scale bar: 20 μm. All images were taken with a Zeiss 40× 1.3 oil objective. **f** Top: Representative single-channel current recordings obtained from the released protoplast at *V*_*h*_ of +20 mV and 0 mV. o: open channel current level; c: closed channel current level. The calibration bar is on the right side. Bottom: current-voltage (IV) dependency of the recording shown above. On the abscissa, the reversal potentials of the main ions in the bath and pipette solutions are shown, indicating that the current is carried mainly by glutamate. The obtained conductance (*g*) is given above the linear fit through the measured points. Recorded in SolB. **g** Representative current recorded from the entire protoplast membrane in the whole-cell configuration. The cells were recorded in SolA with a low-chloride pipette solution. The voltage stimulation protocol used to obtain the recordings is shown in the inset. The calibration bar is at the bottom

A nonlinear laser scanning microscope (NLSM) using fs laser pulses is the necessary prerequisite for the application of precision laser ablation. The NLSM system also enables in vivo imaging of single cells and cellular structures using two-photon excitation fluorescence (TPEF) before and after laser nanosurgery. We successfully created laser-generated incisions in cell wall sections of the filamentous fungus *Phycomyces blakesleeanus* using tightly focused, 160 fs, near-infrared (NIR) laser pulses (Fig. 1). To remove a small portion of the cell wall in a specific area of the hyphae, the cell wall must be visualized and distinguished from the plasma membrane and other cell structures. Since the cell wall and the plasma membrane are in close contact^46^, hyphae were plasmolyzed and kept in hyperosmotic solution to retract the cytoplasm before the procedure, as shown in Fig. 1a. The plasmolyzed hyphae stained with calcofluor white (CFW) and treated with an exocytosis inhibitor and a respiratory inhibitor (sodium azide) to prevent cell wall regeneration were subjected to nanosurgery. We selected for the nanosurgery the section where the hyphal protoplast was retracted from the cell wall and placed the spot-wise pattern on the selected section (Fig. 1b). The laser-generated incision is clearly visible in the TPEF images (Fig. 1c) and in the bright-field images. The cell viability after nanosurgery was regularly verified by bright-field microscopy. The protoplast enveloped by the plasma membrane exited through the incision in the cell wall without deforming or bursting (Fig. 1d). Thus, the released round protoplasts were viable for up to several hours during the electrophysiological measurements. Representative patch-clamp current recordings obtained from the released protoplasts in the “outside-out” and “whole-cell” configurations are shown in Fig. 1f and g, respectively.

We obtained 79 fungus-released protoplasts in the patch-clamp microscopy chamber that were sufficiently large (larger than 7 μm) and spatially accessible to the patch pipette. Membrane contact with high resistance (greater than GΩ resistance) was obtained on 22 released protoplasts (28%). Eight protoplasts (10%) were very stable and allowed multiple successful pipette approaches which resulted in the formation of multiple GΩ-resistance contacts and patch excisions. A total of 36 giga-ohm seal contacts were obtained. All patch-clamp configurations were used for the recording. The current family obtained from the entire protoplast membrane in the “whole cell” configuration had a typical appearance (Fig. 1f). The single-channel currents recorded from the excised patches in the inside-out or outside-out configurations were more diverse. We used solutions with asymmetric ion concentrations to identify the general type of ion basis for selectivity (K^+^, Cl^−^, glutamate^−^, or Ca^2+^) for each recorded channel current based on the reversal potential (*V*_*rev*_) (Fig. 1g).

### Laser nanosurgery procedure—the main steps and key factors

**Fig. 2:**
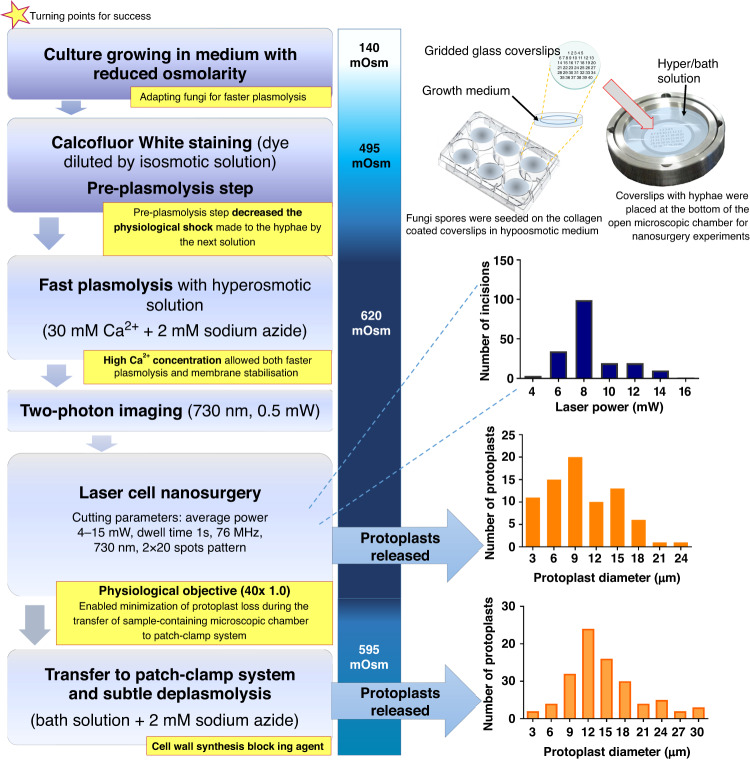
Laser nanosurgery procedure main steps and turning points for successful removal of the *Phycomyces blakesleeanus* hyphae cell wall. The osmolarity of the extracellular solution at each step of the procedure is indicated on the blue gradient strip. A detailed description of the procedure can be found in the Materials and Methods section. The distribution of the average laser power used for nanosurgery of *P. blakesleeanus* cell walls is shown in the upper graph, and the distributions of the sizes of the protoplasts released in the two consecutive steps (bottom) are shown as histograms with the upper bin boundary on the abscissa

The main steps for successful fs laser nanosurgery of the fungal cell wall to obtain viable protoplasts for patch-clamp analysis are shown in Fig. 2. The first important step was to decrease the osmolarity of the growth media to acclimate the fungi to conditions with lower osmolarity and increase their sensitivity to the plasmolysis solution. This approach ensured faster plasmolysis during the preparation phase of nanosurgery. By reducing the osmolarity of the growth medium by 30%, the plasmolysis time (in minutes) was decreased from 30 ± 10 to 5 ± 2. The introduction of two-stage plasmolysis and the increase in the Ca^2+^ concentration in the hyperosmotic solution followed, and these were considered the most important, key factors for successful surgery and the recovery of viable, patchable, released protoplasts. The Ca^2+^ concentration was increased to accelerate plasmolysis (<1 min) and stabilize the protoplasts^33^. The step before plasmolysis reduced the physiological shock that would occur if the fungi were transferred directly from the medium to a hyperosmotic solution (450 mOsm difference). This was achieved by staining the fungi with diluted dye in a standard isoosmotic solution.

To achieve precision and enable successful nanosurgery and imaging, the fungal hyphae were immobilized on the coverslip. A number of substrate-coating materials were tested (Supplementary Table 1), and a collagen coating was found to provide better immobilization of the fungal hyphae while allowing for the patch-pipette approach.

The cell wall of the filamentous fungus *Phycomyces blakesleeanus* is weakly autofluorescent and can be visualized by excitation at 730 nm, presumably as a signal from the chitin-containing (chitin and chitosan) component of the cell wall^47,48^. The removal of the cell wall by the laser nanosurgical procedure was not efficient enough on the native hyphae. After staining the cell wall with CFW, the surgery was almost 100% successful. All nanosurgical procedures presented were performed with a cell wall stained with CFW to improve the absorption of laser energy. CFW has a high affinity for cell wall components (chitin and other polysaccharides), and the absorption maximum is at 345 nm under single-photon excitation conditions^49^.

Two-photon imaging of the cell wall was performed with a very low average laser power of 0.5 mW and a pulse energy of 0.007 nJ at the sample plane. A wavelength of 730 nm was used for both multiphoton imaging and nanosurgery, bypassing the time required to change wavelengths. A diameter of hyphae (<15 μm) poses a major challenge for successfully cutting off a portion of the cell wall without endangering the rest of the cell. After optimizing the surgical procedure, we selected a 2 × 20 spot-wise pattern positioned on the cytoplasm-free section of the cell wall in the TPEF image. Depending on the degree of plasmolysis, i.e., the length of the site available for cutting, the 2 × 20 dot pattern was scaled (adjusted: scale, angle, and position) for surgery to the size that would cut off as much of the wall as possible without critically approaching the plasma membrane. Based on the selected pattern length, cell wall thickness, and proximity to the protoplast, the cutting power was selected in the drawing mode of the software. We were able to control the parameters of surgery: dwell time per point, laser power and wavelength. The surgical procedure was automated, started “on click” and lasted approximately 20 s. Prior to the surgical experiments, tests were performed to determine the average laser power required to cut a small portion of the cell wall, and the damage threshold was determined. The damage threshold was approximately 3.5 mW (using a 40× 1.0 physiological objective). The overall distribution of laser powers at the sample plane used for nanosurgery is shown in the upper graph in Fig. 2; the lower power range was used in the majority of the operations. Laser powers of 6 to 12 mW were used in 90% of the cell wall operations. The dwell time of 1 s per point was maintained at the same value during the surgical procedure for all hyphae. Femtosecond pulses (160 fs) at a 76 MHz repetition rate and very low pulse energies (0.05-0.20 nJ) at the specimen plane ensured successful wall cutting with high spatial resolution of the surgical incisions.

Subsequently, subtle deplasmolysis was performed to stimulate the protoplast release without overstretching the membrane to interact with the pipette. To avoid hypoosmotic stress and maintain the integrity of the protoplast plasma membrane, the osmolarity of the solution in the microscopic chamber was reduced by only a few tens of mOsm such that slightly hyperosmotic conditions were present. The optimized concentration of sodium azide for inhibiting cell wall production was consistently present in all solutions, and its presence was an indispensable factor for success. Respiration and growth of *Phycomyces blakesleeanus* under the conditions used for nanosurgery are shown in Suppl. Fig. S1.

Because the nanosurgery (using an upright nonlinear microscope) and patch-clamp (using an inverted microscope) systems are separate, the physiological objective played a key role. Since a dip-in objective is normally used without a top coverslip, it was possible to approach the sample above and completely immerse the objective tip in the solution surrounding the sample. Consequently, there was no need for removal of the coverslip, which would otherwise lead to possible loss of the emerged protoplasts. On the other hand, the gridded coverslips on which the fungi were grown and placed at the bottom of the open microscopic chamber allowed the precise localization of the hyphae subjected to nanosurgery.

As expected, the size of the patch pipette influenced the success of giga-ohm sealing. For our purposes, the best pipettes for the fungal protoplast patch-clamp were between 10 MΩ and 15 MΩ. Smaller and larger pipettes were two times less likely to achieve good-quality contact. The protoplast size was also an important factor in the success of high membrane-pipette contact formation. When contact was successful, the released protoplasts were significantly (*p* = 0.016) larger (18 ± 6 µm) than those where contact (15 ± 5 µm) could not be achieved. Potentially, with further practice, the success rate of achieving contact with the gigaohmic seal could be significantly improved for these smaller released protoplasts. To estimate the success rate of patch-clamp contact on released protoplasts that were sufficiently large and accessible to the pipette, we divided the number of giga-ohm contacts obtained by the number of patch-pipette contacts attempted on “patchable” protoplasts. Overall, the success rate was 55%, with 23 successful contacts out of 42 pipette approaches. The entire process (cell surgery + patch clamping) was quite complex, and certain steps needed to be precisely followed for a high success rate and reproducibility. The concentration of chemicals, osmolarity of the solutions, timing and cutting parameters needed to also be kept within a specified narrow range.

### Electron microscopic imaging of the surgical incision in the cell wall and released protoplast

To verify the appearance of the edges of the incisions in the plasmolyzed hyphal cell wall, we imaged the fungus subjected to nanosurgery using a scanning electron microscope (SEM).

**Fig. 3:**
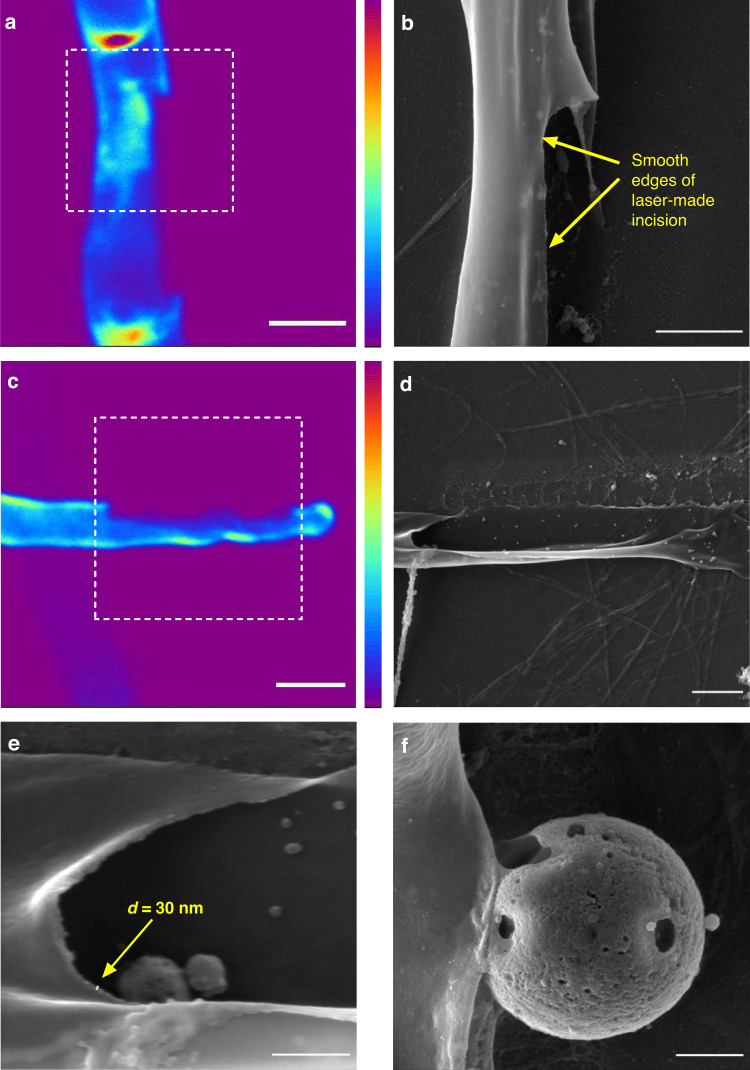
SEM images of the *Phycomyces blakesleeanus* cell wall after the femtosecond laser nanosurgery. **a** TPEF image of the fs laser-generated incision in the hyphal cell wall. The average laser power in the sample plane was 1.0 mW for imaging (dwell time 2.5 µs) and 7.5 mW for surgery (dwell time 1 s). Scale bar: 5 µm. **b** SEM image of the same laser-generated incision shown in a. Scale bar: 2 µm. **c** TPEF image of the fs laser-made incision. The average laser power in the sample plane was 1.0 mW for imaging (dwell time 2.5 µs) and 7.6 mW for surgery (dwell time 1 s). Scale bar: 5 µm. All TPEF images were acquired with a Zeiss 40× 1.3 oil objective. The color intensity bars for the TPEF signal are as follows: violet/blue, lowest TPEF signal; and dark red, highest TPEF signal. The color intensity bar is linear and covers the entire range of the data. **d** SEM image of the entire laser-generated incision, same as that shown in c. Scale bar: 2 µm. **e** Hyphal cell wall thickness (*d*) of the incision shown in d. Scale bar: 0.5 µm. **f** Released protoplast through a laser incision made with 6.7 mW average power at 730 nm. Scale bar: 1 µm

As shown in Fig. 3, SEM images revealed crack-free precision cuts, with smooth edges of the slit in the hyphal cell wall made with the femtosecond laser (*n* = 11 incisions at 10 images). The released protoplast membrane had no large masses of remaining or deposited material (*n* = 2) (Fig. 3f). The surgical parameters used allowed localized removal of the cell wall with an average thickness of 30 nm (Fig. 3e), sometimes even at extremely short distances from the plasma membrane (0.5–3 μm), leaving the released protoplast viable. The mean length of the incisions measured from TPEF images was 6.7 ± 2.9 µm (*n* = 185), whereas mean length of the incisions measured from the SEM images was 9.3 ± 4.4 µm (*n* = 6). The width of the incisions measured from the SEM images was 2.2 ± 0.2 µm (*n* = 3).

### Protoplast release parameters

**Fig. 4:**
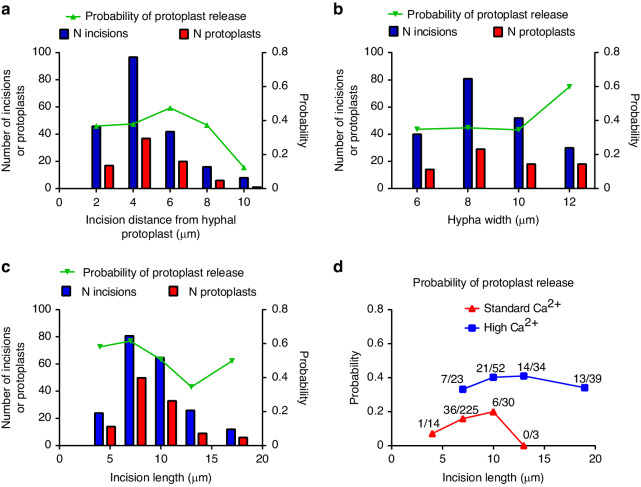
Parameters affecting the probability of protoplast release. Probability of protoplast release from the nanosurgery-produced incision, superimposed on the corresponding histogram bin of parameter values. **a** Distance of the laser incision from the hyphal protoplast during nanosurgery: distribution and dependence of the probability of protoplast release. **b** Hyphal width at the site of nanosurgery: distribution and dependence of the probability of protoplast release. **c** Length of surgical incision: distribution and dependence of the probability of protoplast release. **d** In a separate series of experiments, the effect of increased calcium concentration on the probability of protoplast release was measured as a function of incision length in the nanosurgical phase of the procedure, without subsequent steps of deplasmolysis. High [Ca^2+^]: 30 mM, as used in the surgical protocol. The hyperosmotic solution used was 600-617 mOsm throughout the protoplast release period. Standard [Ca^2+^]: 3 mM and 1 mM (pooled data). The hyperosmotic solution used was 555-560 mOsm throughout the protoplast release time. For each 3 µm incision size bin in the histogram, the corresponding number of protoplasts obtained divided by the number of surgical sections performed is shown above the probability curve. In all graphs, the abscissa represents the upper limit of the bin. For **a**–**c**: *n*_*tot*_(incisions) = 203 (**a**); 209 (**b**); 208 (**c**). *n*_*tot*_(protoplast) = 71(**a**), 81(**b**), 112 (**c**). For **d**: *n*_*tot*_(incisions) = 272 (Standard Ca^2+^); 148 (High Ca^2+^). *n*_*tot*_(protoplast) = 43 (Standard Ca^2+^); 55 (High Ca^2+^)

After successful creation of the surgical incision in the cell wall, the release of protoplasts into the surrounding medium was observed. Not all incisions released protoplasts. We found that several factors influenced the probability of protoplast release (*Pr* = number of protoplasts/number of incisions). The distance of the laser cut from the protoplast in the hypha during the procedure influenced how many protoplasts could be recovered. As shown in Fig. 4a, when the protoplast in the hypha was more than 6 µm from the incision site, the probability of protoplast release decreased from approximately 0.4 and was lowest at the largest distances tested. This result further confirmed that the protoplasts were not damaged in our protocol, even when the site of nanosurgery was in close proximity to the retracted hyphal protoplast. The width of the hyphae (*w*_*h*_), which reflected the overall size of the hyphae, did not appear to affect the possibility of obtaining released protoplasts for *w*_*h*_ < 10 µm. Wider hyphae (*w*_*h*_ > 10 µm) were more favorable for protoplast release with a *Pr* of 0.6, while for smaller hyphae, *Pr* was 0.35 (Fig. 4b). Finally, the size of the incision also affected the release probability, with smaller incisions releasing more protoplasts. As shown in Fig. 4c, the probability decreased with increasing incision length, from *Pr* = 0.62 at an incision length of 5.5 ± 1.5 µm to *Pr* = 0.35 at an incision length of 11.5 ± 1.5 µm. We are confident that all hyphae were viable after the nanosurgical procedures because they retained their characteristic cytoplasmic streaming and healthy appearance and were deplasmolyzed when transferred to less hyperosmotic conditions. Thus, the dependence of *Pr* on incision size was caused by the influence of underlying physiological or mechanical aspects controlling protoplast release. We also noted a slightly better protoplast yield in the plasmolyzed hyphae that exhibited a cytoplasmic bridge across the “empty” region near the incision site than in those whose hyphal protoplasts were completely separated (*Pr* = 0.44 and 0.36, respectively).

In a separate set of experiments on nanosurgery, we examined protoplast release immediately after nanosurgery, without a deplasmolysis step, under slightly different plasmolysis and surgical conditions, as explained in the Methods section. We investigated whether the high calcium concentration used throughout the procedure affected protoplast release (Fig. 4d). We compared protoplast release at a high Ca^2+^ concentration (30 mM) with protoplast release at a standard Ca^2+^ concentration (1 and 3 mM, merged). At standard Ca^2+^ concentrations, the probability of protoplast release was clearly dependent on the size of the incision and was quite low ( < 0.2). Increasing [Ca^2+^] to 30 mM increased the probability of obtaining the protoplast for the same incision size to 0.4. The calcium dependence of the probability of protoplast release indicated that a physiological mechanism was involved in the process of expelling protoplasts from the hyphal wall through a surgically made incision.

### Laser nanosurgery of the cell wall at different sites along the hyphae

**Fig. 5:**
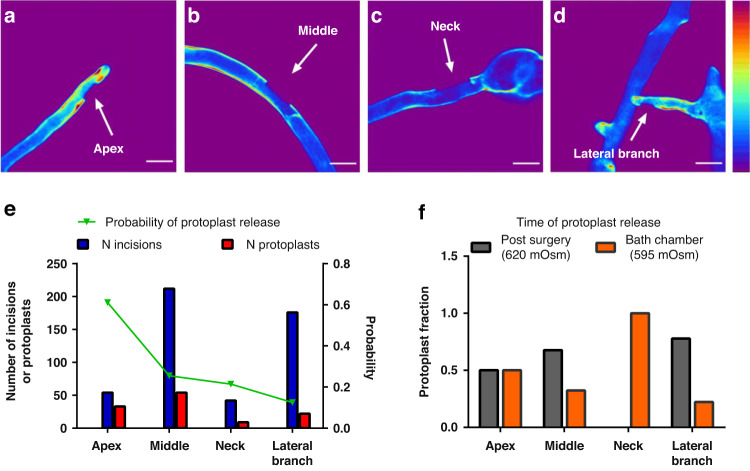
Nanosurgery applied to different sites on the hyphae. TPEF images of the hyphae with a laser surgical incision at different locations: **a** apex, **b** middle, **c** “neck” and **d** lateral branch of the labeled hypha. The white arrows indicate a laser-made incision in the hyphal cell wall. All images were taken with a Zeiss 40× 1.3 oil objective. The color intensity bars for the TPEF signal are as follows: violet/blue, the lowest TPEF signal; and dark red, the highest TPEF signal. The color intensity bar is linear and covers the entire range of the data. Scale bar: 10 μm. **e** Probability of the protoplast release at each site (apex, middle, neck and lateral branch), with the corresponding number of incisions obtained. **f** Osmotic conditions during the protoplast release at the different sites are shown as fractions of the total number of protoplasts released at each site in each of the solutions used: “postsurgery”= 620 mOsm; “bath chamber” = 595 mOsm

Since hyphae are polarized cells, selective sampling from different hyphal regions would be highly desirable. We exploited the precision and flexibility of the nanosurgical method to perform incisions and obtain protoplasts from the localized portions of the following hyphal regions (“compartments”): 1. The tip (apex), which represents the fast-growing portion of the hyphae (Fig. 5a); 2. The middle (Fig. 5b); 3. The “neck”, which represents the base of the hyphae, just below the spore (Fig. 5c); And 4. The lateral branch (Fig. 5d). The average laser power used for the cuts in the different compartments did not vary, while the distance of the incision from the hyphal protoplast at the time of nanosurgery needed to be adjusted due to the narrower space in the side branch compartment; the distance of the incision from the hyphal protoplast was shorter in the lateral branch compartment than in the middle compartment: *p* = 0.0220; compared to the tip compartment: *p* = 0.0003. Figure 5e shows the number of incisions with their *Pr* values for each site. The *Pr* was highest in the apical region (0.6) but rather low in the lateral branches (0.13). Interestingly, the protoplasts were released from the different regions under different osmotic conditions (Fig. 5f). For example, protoplasts from the neck did not appear in the hyperosmotic solution used for surgery, with 100% of protoplasts obtained from the neck were released during deplasmolysis in the bath chamber; however, protoplasts from the hyphal tip were released equally well in both hyperosmotic and deplasmolysis solutions. The success in achieving giga-ohm contact was highest for protoplasts from lateral branches (55%), while it was 25-30% for protoplasts from other regions.

### Registered currents of protoplasts obtained by nanosurgery

**Fig. 6:**
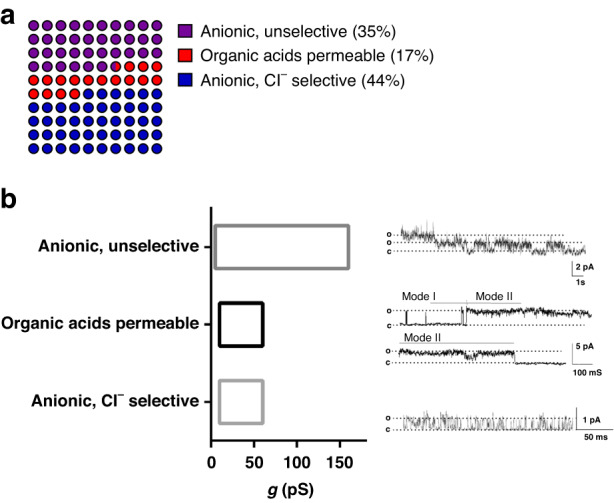
Summary of the properties of the obtained currents. **a** Major types of currents present in the protoplast membrane categorized according to ion permeability. Ion permeability was determined from IV plots under asymmetric conditions, as shown in the bottom panel of Fig. 1g. **b** Conductance ranges determined for major protoplast types of ion currents, with representative current recordings shown on the right. Unselective anionic current recording, inside-out, *V*_*h*_ = +80 mV, bath solution with high glutamate content (solB), *g* = 15 pS; Organic acid-carried current, outside-out, *V*_*h*_ = +30 mV, bath solution with nitrate (solA), *g* = 40 pS, activity modes indicated above current recording. Cl^−^ -selective current, outside-out, *V*_*h*_ = +80 mV, solB, *g* = 12 pS. o: open channel current level; c: closed channel current level. The calibration bar for each recording is presented on the right side

The excised patch currents measured from protoplasts obtained by nanosurgery were predominantly anionic. Only in one case did the measured current have a reversal potential corresponding to calcium ions. Approximately one-third (35%) of the registered currents belonged to the group mediated by nonselective anionic channels that transport both chloride ions and organic acids (“anionic unselective”), whereas 44% of the currents were mediated by channels that are highly selective for chloride (“Cl^−^ selective”) (Fig. 6a). Some of the currents recorded (17%) were mediated by channels highly permeable to glutamate (“organic acids permeable”). These currents had a reversal potential close to the calculated *V*_*rev*_ for glutamate. Figure 6b shows the total conductance range (g) for each group, along with typical excised patch current recordings. Most frequently, Cl^−^ selective currents had short openings and closings at depolarized potentials (*n* = 7) (Fig. 6b bottom trace), whereas glutamate-permeating currents alternated between two modes: (I) infrequent and relatively short openings and (II) several hundred ms long openings with sub-conductance levels interspersed with very short closed states (*n* = 4). Figure 6b middle trace shows the current that started in mode I and then abruptly transitioned to mode II. Nonselective currents exhibited a wide range of conductances and likely represent a heterogeneous group. Top trace in Fig. 6b shows the most frequently recorded current activity, bursts of current accompanied by very short needle-like openings (*n* = 7).

## Discussion

In this study, we described a highly reproducible laser-based technique for the nanodissection of the fungal cell walls to obtain released viable fungal protoplasts whose plasma membrane was suitable for patch-clamp electrophysiology. Similar approaches were reported long ago using ns-UV laser pulses but were not further developed^10,33^. Compared with ns lasers, fs lasers can target specific regions with high accuracy without damaging the out-of-focus region, resulting in better surgical outcomes. They can produce clean and precise incisions with minimal thermal or mechanical damage. The high precision of fs laser ablation enabled the cutting of a small piece of cell wall without endangering the plasma membrane of the protoplast; this cut was only a few micrometers away from the protoplast or even closer. Another advantage of fs IR lasers over ns UV lasers is the wavelength used. IR lasers have longer wavelengths than UV lasers; thus, they can penetrate deeper into tissues due to nonlinear two-photon absorption that occurs only in the focal volume^38^. UV lasers that emit shorter wavelengths have more energy, increasing the susceptibility to cellular DNA damage^50^ and inducing oxidative stress and cell death^51^. Another advantage of this method is the use of the same fs IR nanosurgical laser for two-photon imaging of live hyphae to mark the desired cell wall area for surgery. Compared to previous studies in which bright-field images were used to mark and section the cell wall, in the present study, we used two-photon fluorescence of the cell wall from TPEF images. The ability to select a region on the TPEF images for cutting contributed greatly to the accuracy and precision of the overall cell surgery method^38^. We did not observe any vaporization, cavitation, or carbonization on the TPEF or BF images recorded after the surgical procedure. Imaging the specimen by either modality during the surgical action was not possible. Additionally, in the SEM images, we did not observe fragments of the removed cell wall. The processes that potentially occurred during surgery and the possible transformations of the ablated/ejected materials are described in detail in Vogel and Venugopalan 2003^52^. The nanosurgery method presented here could lead to successful patch-clamp recording only if the conditions for each step were kept within the optimal range, as many factors influenced the biological aspects of the procedure.

Fungi exhibit a cell wall integrity response; this is a signaling pathway activated by cell wall stress or damage involving fungal-specific kinases^53^ and is the basic survival mechanism. The cell wall integrity pathway is involved in the response to osmotic stress^54^, fungal morphogenesis, and fungal pathogenicity. The respiratory inhibitor azide was used throughout the protocol because it is the only respiratory chain inhibitor capable of almost complete inhibition of classical respiration for prolonged periods (unlike cyanide^55^) without significant induction of alternative respiration (unlike antimycin^55^); its use resulted in a long-term reduction in total respiration to 20% while maintaining viability. Under conditions of this pronounced metabolic suppression, all intracellular signaling pathways that depended on kinase activity and therefore required ATP, including the cell wall integrity response, were assumed to be suppressed.

Prevention of new wall formation was one of the key elements in the procedure’s success because when azide was omitted, the hyphal protoplasts remained in the hyphae. In rare cases, when a protoplast was released without an applied azide due to the vigorous hypoosmotic swelling immediately after producing the wall incision, the contact between the patch pipette and the protoplasts was not achieved. The high concentration of calcium used throughout the procedure could account for the complete absence of potassium permeating currents in our system. The fungal-specific tandem pore outwardly rectifying K^+^ channels (TOKs) are found in the genome of filamentous fungi, and heterologous expression shows that they form functional K^+^ channels that may be present in the plasma membrane of filamentous fungi^12,56^. The TOK channels are blocked by Ca^2+^ on the extracellular side of the membrane^12^. Therefore, the almost absolute dominance of anionic currents (96% of all recorded currents) could be a result of the experimental conditions. Furthermore, the hyperosmotic conditions during the procedure could influence the activation or properties of the registered ionic currents. Notably, the osmolarity of the extracellular solutions used during patch-clamp seal formation and electrophysiological recordings was approximately isosmotic (with respect to the cytoplasm) because the protoplasts were optimally “inflated”; otherwise, the membrane would not be suitable for sealing. Filamentous fungi are organisms adapted to a wide range of different environmental conditions, such as the osmolarity of the surrounding environment; therefore, the possible change in physiology caused by a shift in osmolarity is likely to be the biologically relevant physiological state^57^.

Immediately after surgery, protoplast release was frequently observed, and the probability of protoplast release was higher when the wall incision was made in close proximity to the hyphal protoplast. Turgor pressure-induced release of protoplasts could not be ruled out as part of the explanation for this phenomenon since the pressure relative to the cell wall in intact hyphae is 500 kPa greater, providing turgor to the cell^58,59^. However, under hyperosmotic conditions, turgor pressure is lower^58^, and an additional cellular process, likely mediated by the cytoskeleton, could be involved in the protoplast release, since an increased calcium concentration enhanced the protoplast release from the incision. The more successful formation of sealing contacts in the protoplasts released from side branches was potentially due to the favorable interaction with the young plasma membrane, as is often the case with enzymatically derived plant protoplasts^24^. Notably, the close proximity of protoplasts to the laser incisions during nanosurgery had no detrimental effect on the health of the plasma membrane; this was reflected by the ability of the protoplasts to make high-resistance contact with the patch pipette, further demonstrating that the protocol presented in this study could produce consistently healthy released protoplasts from versatile locations. Our finding that numerous anionic conductances are present on the plasma membrane of filamentous fungi is consistent with the data from a limited number of available cloned ion channels^13,15^ revealing the presence of CLC-like and Bestrophin-like channels. Additionally, unidentified ion channels could be present on these membranes. The prospects for further applications of the presented method include the discovery and identification of ion currents in the native protoplast membrane that have remained undetected to date because of the low homology of the underlying channels with animal or plant counterparts. These advances would further the understanding of the physiology of filamentous fungi.

*Phycomyces blakesleeanus*, the filamentous fungal model system used in this work, is a member of the order Mucorales (representative of the phylum Mucoromycota); members of this order form mycorrhizal symbiotic relationships with plants^60^, while other members are serious human pathogens^61^ whose prevalence is increasing^62,63^. The recently published WHO recommendation^64^ identifies Mucorales as a high-priority group for monitoring, researching, and developing new drug targets. Therefore, the discovery and characterization of the membrane currents of *Phycomyces blakesleeanus* involved in the physiological responses of hyphae could lead to the identification of much-needed novel drug targets to combat human pathogens^65^, as well as to better protect or exploit the mycorrhyzal symbiosis with plants in ecosystems and agriculture^66^. Further modifications of the described method may be required in the future to expand its application to other filamentous fungal phyla. The potential applications of this technique span the entire range of fungal biology applications.

## Materials and methods

### Culture and growth conditions

A wild-type strain of the filamentous fungus *Phycomyces blakesleeanus* (Burgeff) [NRRL 1555(-)] was used in this study. The spores were seeded at a concentration of 10^6^ spores/mL in standard liquid minimal medium (SLM)^67^ at pH 4–5 containing the following per liter: 2 g L-asparagine·H_2_O, 5 g KH_2_PO_4_, 500 mg MgSO_4_·7H_2_O, and microelements (28 mg CaCl_2_, 1 mg thiamine hydrochloride, 2 mg citric acid·H_2_O, 1.8 mg Fe(NO_3_)_3_·9H_2_O, 1 mg ZnSO_4_·7H_2_O, 300 µg MnSO_4_·H_2_O, 50 µg CuSO_4_·5H_2_O, and 50 µg Na_2_MoO_4_·2H_2_O), with half the glucose concentration (10 g D (+)-glucose) (1/2glucSLM) to reduce the osmolarity of the growth medium. The osmolarity was additionally decreased by diluting the medium by 10% from 210 to 140 mOsm to adapt the fungus to lower osmolarity conditions and ensure faster plasmolysis during the preparatory phase of nanosurgery. Fungi were grown in illuminated stationary open 100 mm Petri dishes at 21-22 °C.

### Sample preparation

For cell nanosurgery experiments, the fungi were grown on glass coverslips (*d* = 15 mm) coated with a thin layer of 50% type I collagen as an immobilizer and placed on the bottom of 6-well plates. The collagen was previously polymerized at room temperature for 24 hours. The collagen-coated coverslips were inoculated with 15 µL of spore suspension from a Petri dish culture with an additional 50 µL of diluted 1/2glucSLM medium (to prevent the sample from drying out); the sample was allowed to stand for 1 hour to allow the spores to adhere/anchor to the collagen. After 1 hour, 2 mL of diluted 1/2glucSLM medium was added to the wells. This method proved to be the best in terms of hyphal adherence and growth. The experiment was always performed with fungi from the exponential growth phase (19-30 hours old). Most of the fungal hyphae were simple, elongated cells with a “spore “head” still visible, although some hyphae had just begun to form side branches.

Previously, we performed hyphal adhesion experiments on the following substrate-coating materials and coverslip surface modifications: gelatin, silicone, laminin, poly-L-lysine, concanavalin A, collagen type I, and plasma treatment (Supplementary Table S1). The hyphae adhered to and grew best on collagen type I. The optimal collagen thickness was then determined and found to be 10 μL of working collagen solution on a 15 mm diameter coverslip. Half-millimeter numbers were engraved on the coverslips (with a diamond scribe pencil) before the collagen treatment. Mapping of the culture coverslips was necessary to accurately determine the positions of the fungal hyphae before and after surgery because the surgery itself and the electrophysiology were performed on two separate microscopes.

Before the experiments, a coverslip containing the immobilized hyphae was placed on the bottom of the microscopic chamber and filled with the appropriate amount of hyperosmotic solution.

### Staining of the cell wall

Live hyphae from the exponential growth phase (19-30 h) were incubated with 1% Calcofluor White (CFW; Sigma Aldrich) dye in isosmotic solution (495 mOsm) for 10 min at 20 °C. Then, 10 μM brefeldin A was added to the isosmotic dye solution. After incubation, the hyphae were washed three times in minimal media.

### Preparation of solutions

The isosmotic solution for the CFW step contained (in mM) 60 KCl, 65 K-glutamate, 2 MgCl_2_, 1 CaCl_2_, and 10 HEPES (pH 7) supplemented with sucrose to produce 495 mOsm.

The hyperosmotic extracellular solutions (hyperECSs) used for plasmolysis and during nanosurgery contained the following (in mM): 60 KCl, 65 K-glutamate, 2 MgCl_2_, 30 CaCl_2_, and 10 HEPES at pH 7 supplemented with sorbitol to achieve the desired osmolarity (620 mOsm). In some experiments, slightly more hyperosmotic conditions were used (hyperECS: 680 mOsm, bath solution: 640 mOsm). Of the 13 attempts at the pipette approach, only 2 were successful using these conditions.

In a subset of experiments, the solution was modified to match the ion content of the patch-clamp bath solution and to investigate the ion basis of the recorded currents. For experiments with nitrate-based solution, hyperECS was modified by replacing CaCl_2_ with Ca(NO_3_)_2_. For experiments with glutamate-based solutions, the ratio of KCl to K-glutamate in hyperECS was changed to 1:4 or 1:0, while the sum of the concentration remained at 125 mM.

For the experiment on the effect of Ca^2+^ on protoplast release, solutions with the following composition were used (in mM): standard [Ca^2+^] solution (1 mM and 3 mM; data were pooled) - 60 KCl, 65 K-glutamate, 2 MgCl_2_, 10 HEPES, 1 or 3 CaCl_2_, pH 7, supplemented with sorbitol to 555-560 mOsm.

The high [Ca^2+^] solution had the same ionic composition as the standard Ca^2+^ solution; the only differences were CaCl_2_ (30 mM) with an osmolarity of 600-617 mOsm.

### Patch-clamp recording solutions

The osmolarity of the chamber bath solution was 595 ± 10 mOsm; this solution was 25 mOsm hyperosmotic than the pipette solution (570 mOsm). During the deplasmolysis step with a solution change, appropriate solutions were used to alter the ion composition of the final solutions to the following: nitrate solution (SolA) (in mM): 82.75 Cl^−^, 125 K^+^, 48.75 glutamate, 23.75 Ca^2+^, 45 NO_3_^−^, 2 Mg^2+^, 10 HEPES; and high-glutamate solution (SolB) (in mM): 70.25 Cl^−^, 125 K^+^, 106.25 glutamate, 23.75 Ca^2+^, 2 Mg^2+^, and 10 HEPES. The patch pipette solutions contained the following (in mM): solution combined with SolA: 125 KCl, 5 CaCl_2_, 2 MgCl_2_, and 10 HEPES; low chloride solution: 50 KCl, 5 K-glutamate, 1 CaCl_2_, 2 MgCl_2_, and 10 HEPES. The following patch pipette solution was used in combination with SolB: 125 KCl, 5 K-glutamate 1 CaCl_2_, 2 MgCl_2_, and 10 HEPES. All solutions were sterile-filtered at pH 7.

### Hyphal plasmolysis and deplasmolysis

Plasmolysis was performed in two steps. In the pre-plasmolysis step (10 min incubation), hyphae were transferred from hypoosmotic growth medium to an isosmotic solution (495 mOsm) containing the cell wall dye. This step prior to plasmolysis served to facilitate the transition of the hyphae into the hyperosmotic solution used during imaging and nanosurgery. The hyperosmotic solution (HoS) (620 mOsm, except for one subset in which 680 mOsm was used) contained a high concentration of calcium (30 mM) in the form of CaCl_2_ or Ca(NO_3_)_2_. A high Ca^2+^ content enabled faster plasmolysis and stabilization of the membrane by increasing the ionic strength of the hyperosmotic solution.

The onset of plasmolysis was considered the time at which hyphal protoplast withdrawal was observed at two or more sites along the hyphae in at least 25-30% of the hyphae in the field of view. Retraction from the tip was not used as a criterion for measuring plasmolysis time. After the nanosurgical procedure, the gentle deplasmolysis step was performed. We carefully removed ¼ of the hyperosmotic solution and slowly added 520 mOsm solution in the same volume, which decreased the osmolarity of the solution in the microscopic chamber to 595 ± 10 mOsm. A difference of 25 ± 10 mOsm between hyperECS and the bath solution allowed protoplasts to exit in some cases. However, in the majority (64%) of the experiments, protoplasts exited the hyphal wall through the laser-generated incision while still in the hyperosmotic solution.

Fungi were treated with an exocytosis inhibitor (10 μM brefeldin A) added before the plasmolysis step and a respiration inhibitor (2 mM sodium azide) to prevent cell wall regeneration during the procedure. Sodium azide was continuously present in all solutions (except during CFW staining). The addition of enzymes (chitinases and chitosanase cocktail) that would breakdown cell wall residues, which we hypothesized might be present at the cut site, was tested; however, these enzymes were found to have no effect and were not used under the optimized conditions.

### Measurement of the biomass yield and oxygen consumption

To measure the biomass yield, *P. blakesleeanus* mycelia were grown in Petri dishes for 16 hours in 500 (control), 760 and 860 mOsm solutions. The biomass was measured after 30 and 90 minutes for both the control and treatment groups. The yield gain was determined for each culture as the biomass at each time point reduced by the average biomass of the control at the beginning of growth. The growth inhibition percentage was calculated as follows: (yield gain/average of control) *100. Oxygen consumption by *P. blakesleeanus* mycelia was measured using a Clark-type oxygen electrode (Qubit Systems). The mycelial suspension was diluted in fresh liquid minimal medium to an appropriate concentration (oxygen consumption between 15-25 µmol/L × min) and aerated for 1 minute before measurement. Two milliliters of the suspension (*n* = 3) was transferred to a 4 mL electrode chamber and maintained at a constant temperature of 25 °C. The long-term effect of incubation in 5 mM NaN_3_ was measured using a 28-hour-old culture.

Graphs can be found in the Supplemental material (Fig. S1).

### Measurement of the effect of calcium on the protoplast release

The effect of Ca^2+^ on the probability of protoplast release from nanosurgical incisions was measured using images taken immediately after the incisions were made. An oil immersion objective was used for this series of experiments. Nanosurgery and imaging were performed in standard Ca^2+^ or high Ca^2+^ solutions without subsequent patch-clamp recording.

### NLSM experimental setup for TPEF imaging and nanosurgery

**Fig. 7:**
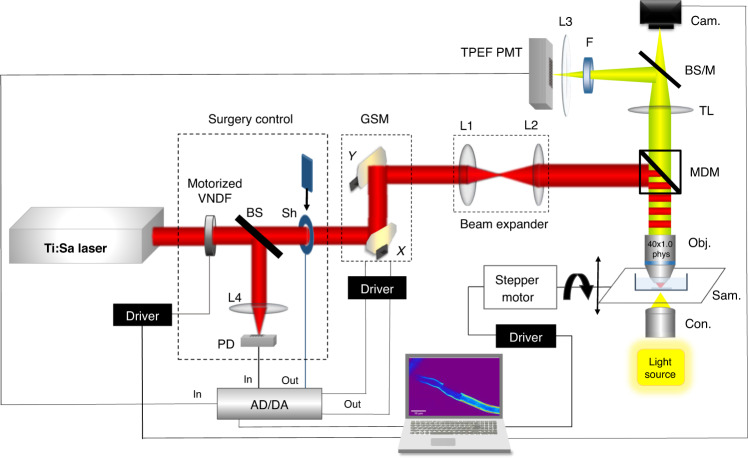
Schematic drawing of the NLSM setup for nanosurgery and cell wall imaging. Ti:Sa fs laser for cell surgery and TPEF imaging, VNDF motorized variable neutral density filter, Sh shutter, GSM galvanometer-scanning mirrors, L1 and L2 - beam expander, MDM main dichroic mirror (cutoff 700 nm), Obj. microscopic objective 40 × 1.0 physiological, Sam. sample, Con. aspheric condenser lens, TL tube lens, BS/M beam splitter or mirror toggle, Cam. camera, F VIS filter 400–700 nm for CFW fluorescence, L3, L4 focusing lens, TPEF PMT photomultiplier tube for TPEF signal, PD photodiode, AD/DA acquisition card

Laser nanosurgery and TPEF imaging of live hyphae were performed via a custom-built nonlinear laser-scanning microscope (Fig. 7) based on the Jenaval upright microscope frame. Previously described in references^47^ and^68^. A femtosecond tunable (700–1000 nm) mode-locked Ti:Sa laser (Mira 900, Coherent, Inc., CA, USA), which generates 160 fs, 30 nJ pulses at a 76 MHz repetition rate, was used. Power control for nanosurgery and imaging was achieved using a motorized variable neutral density filter (VNDF) and fast mechanical shutter (Sh) for blocking or transmitting the laser beam. After passing through the VNDF and shutter, the laser beam was raster scanned over the sample using two galvanometer mirrors (Cambridge Technologies, 6215H; Bedford, Massachusetts, USA). A high numerical aperture (NA) physiological (dip-in) objective lens (Carl Zeiss W Plan-Apochromat 40X, NA = 1.0) was used for tight focusing of the laser beam into the sample. On some occasions, to obtain higher-quality images, we used an oil-immersion high-NA objective lens (Carl Zeiss, EC Plan-Neofluar 40X, NA = 1.3). In this case, the hyphal culture was placed under an additional coverslip on top to enable the use of an oil immersion objective. The physiological objective worked in the same microscopic chamber during laser surgery and patch-clamp procedures on two separate systems. The patch-clamp recordings were performed on a separate system with an inverted microscope that allows the measuring pipette to access the sample from above. A physiological objective lens was used for imaging and surgery on a nonlinear system to perform both procedures of surgery and patch-clamp on the exact same hypha in the same chamber. This process ensured faster transfer from the nonlinear system to the patch system and prevented sample perturbation and possible loss of the obtained protoplast when the upper coverslip was removed. To determine the position for surgery on the hyphal chitin cell wall, it was necessary to visualize the cell wall and ensure that there was sufficient absorption of the laser beam at the wavelength intended for surgery. The intrinsic autofluorescence of chitin enabled imaging of the cell wall. Nevertheless, the hyphae were stained to increase the absorption of the laser beam and consequently reduce the value of the laser power required for sample imaging and nanosurgery. TPEF was used to scan hyphal cell walls stained with CFW dye using the same wavelength as that used for the surgery (730 nm). The fluorescent signal was collected using back reflection by an objective lens, filtered through a visible (400–700 nm) bandpass filter and detected via a photomultiplier tube (PMT) (RCA, PF1006). The steering of the galvo mirrors, the TPEF signal acquisition and shutter were controlled by a USB-6351 National Instrument card at a sampling rate of 1.2 MHz. The power regulation and stage axial position were controlled by a microcontroller. The sample (a coverslip with immobilized hyphae at the bottom of the open microscopic chamber) was placed on the motorized translation stage. The motorized stage was powered by a stepper motor that could translate the sample along the z-axis with 0.3 μm resolution. This approach was essential for accurately targeting the middle of longitudinal sections of the cell wall.

A custom-made add-on to the imaging software was used for the surgical procedure. This approach enabled arbitrary pattern inscription onto the TPEF image, where the dwell time and the average power of the laser beam could be controlled. The surgical procedure was as follows: first, the TPEF image of the hyphae was recorded; second, the pattern (usually the BMP image) was imported and superimposed onto the TPEF image; and third, the scale, exact position and rotation angle were adjusted. Afterward, the average laser power and the dwell time were defined. The shutter was opened at the beginning and closed at the end of the surgical procedure, and then the laser power and the software were automatically set back to the imaging mode. During surgery, the laser beam was moved from point to point for a time (<μs) much shorter than the dwell time (order of 1 s).

Bright-field images were taken by a Canon EOS 50D digital camera (Tokyo, Japan) whose CMOS sensor was placed at the image plane of the tube lens. The BS/M Toggle switch enabled the utilization of either a camera for bright-field or TPEF PMT for fluorescence imaging.

### Scanning electron microscopy (SEM)

The characterization of the laser made incision and the released protoplast was performed using a high-resolution field emission gun scanning electron microscope (FEGSEM-Mira3, TESCAN). The samples were prepared for SEM according to the standard protocol with paraformaldehyde fixation followed by drying in a critical point drying chamber (K850, Quorum Technologies, Laughton, UK) as previously described in ref. ^69^. The samples were sputter-coated with 10 nm of gold/palladium using a Quorum sputter coater to make them conductive for SEM analysis.

### Patch-clamp setup and method

The microscopic chamber was mounted on an inverted microscope (Zeiss Axiovert 10, Germany) with a Luis & Newman micromanipulator system. Currents were measured with an EPC8 amplifier (HEKA), digitized at 10 kHz using an Instrutec 1600 interface (HEKA); the currents were low-pass filtered at 3 kHz, recorded, and processed with Pulse software (HEKA). The pipettes used for on-cell and inside-out patch recordings had a resistance of 10-20 MΩ, while those used for whole-cell and out-out recordings were in the range of 5–10 MΩ. The pipettes were pulled from thick-walled borosilicate glass with filament on a Flaming-Brown P97 pipette puller (Sutter Instruments) and polished with a microforge (L/MCPZ 101, List Medical-Elektronic).

The contact resistance between the pipette and the membrane was monitored by continuous testing with square-wave voltage pulses. Prior to immersion in the bath, the pipette was pressurized to approximately 60 mbar to ensure that any debris between the pipette and the membrane was washed away prior to contact. After contact with the membrane, which resulted in an increase in the resistance of several MΩ, the release of positive pressure was often not sufficient for a tight seal, and a slight negative pressure was also applied to pull the membrane into the pipette; this was then released again to prevent the protoplast from being completely sucked into the pipette. Seal formation typically took a few seconds to half a minute. Giga-seal contact was reached when the contact resistance reached or exceeded 1 GΩ. Recording “on cell” was rarely used because it did not provide satisfactory voltage control. The excision of the membrane patch was achieved by moving the pipette rapidly away from the protoplast in the horizontal direction. The membrane current activity in the inside-out or outside-out configuration was recorded in the gap-free mode of the voltage clamp with the range of holding voltages (*V*_*h*_) to measure the amplitude of the active channels and to generate IV plots. Alternatively, the standard voltage clamp protocol was applied when entering the whole-cell configuration: *V*_*h*_ of –50 mV, followed by a series of steps in 20 mV increments, from −110 mV to +110 mV. The duration of each step sequence was 500 ms, with a rest period of 0.5–1 s. Series resistances were not compensated. All experiments were performed at 20 °C.

### Data analysis and statistics

#### Image analysis

Bright-field image analysis of the extruded protoplasts and cell wall incision lengths was performed using ImageJ (W. Rasband, National Institute of Health, Maryland, USA; http://imagej.nih.gov/ij/).

#### Current analysis

The current data analysis was performed using the Pulse and Clampfit software packages (Molecular Devices, USA). The reversal potentials (*V*_*rev*_) for each ion were calculated using the calculator available at https://www.physiologyweb.com/calculators/nernst_potential_calculator.html. The conductance *g* (pS) was extracted as the slope of the current-voltage dependence for each detectable single-channel current recorded at three or more different *V*_*h*_. The current *V*_*rev*_ was extrapolated from the linear IV plot. In rare cases where the current was rectified (nonlinear IV relationship), the nonrectified portion of the IV plot was used for extrapolation. Care was taken to design the solutions such that the *V*_*rev*_ for each of the ions was sufficiently separated from the others to allow rough typing of the currents based on the ion with the largest contribution to the *V*_*rev*_ (defined as the ion whose *V*_*rev*_ was close to the *V*_*rev*_ of the recorded single-channel current).

#### Statistics

Graphing and statistical comparisons were made using Graph Pad Prism software. Boxes of the box and whisker plots are enclosed by the 25th and 75th percentile ranges, respectively, with the line representing the median; whiskers extend to the minimum and maximum values, respectively. Histograms of the incision length and distance of the hyphal protoplasts from the incision were generated from all values in each group with 3 µm binning and plotted with the upper bin boundary on the x-axis. One-way ANOVA with multiple comparisons and Holm-Sidac correction and the unpaired two-tailed *t*-test with Welch’s correction for unequal variances were used to calculate statistical significance. The confidence intervals for statistical significance were 0.05 (*), 0.01 (**), 0.005 (***), and 0.0001 (****). The release probabilities were calculated from all available data. Throughout the manuscript, the data are presented as the mean ± SD.

### Supplementary information

Supplementary information

## Data Availability

All data needed to evaluate the conclusions in the paper are presented in the paper and/or the Supplementary Materials. Additional data related to this paper may be requested from the corresponding authors.
