# Supplementary material for: In vivo femtosecond laser nanosurgery of the cell wall enabling patch-clamp measurements on filamentous fungi
Source: Microsyst Nanoeng. 2024 Apr 7;10:47. doi: 10.1038/s41378-024-00664-x (PMC10999429; doi:10.1038/s41378-024-00664-x)
Supplement: Supplementary file 1 — Supplementary information [file 41378_2024_664_MOESM1_ESM.docx]

Supplementary information

***In Vivo* femtosecond laser nanosurgery of cell wall enabling patch-clamp measurements on filamentous fungi**

Tanja Pajić^1^*, Katarina Stevanović^1^, Nataša V. Todorović^2^, Aleksandar J. Krmpot^3^, Miroslav Živić^1^, Svetlana Savić-Šević^3^, Steva M. Lević^4^, Marina Stanić^5^, Dejan Pantelić^3^, Brana Jelenković^3^, Mihailo D. Rabasović^3^*

^1^Institute of Physiology and Biochemistry, Faculty of Biology, University of Belgrade, Studentski trg 16, 11158 Belgrade, Serbia

^2^Institute for Biological Research “Sinisa Stankovic”, University of Belgrade, National Institute of the Republic of Serbia, Bulevar Despota Stefana 142, 11000 Belgrade, Serbia

^3^Institute of Physics Belgrade, University of Belgrade, National Institute of the Republic of Serbia, Pregrevica 118, 11080 Belgrade, Serbia.

^4^University of Belgrade, Faculty of Agriculture, Nemanjina Street 6, 11080 Belgrade, Serbia

^5^Institute for Multidisciplinary Research, University of Belgrade, Kneza Višeslava 1, 11030 Belgrade, Serbia

| **Substrate-coating materials** | **Adhesive strength** |
| --- | --- |
| Gelatin | **low** |
| Silicon | **medium** |
| Laminin | **no** |
| Laminin + Poly L Lysine | **low** |
| Concanavalin A (ConA) | **low** |
| Collagen type I | **very high** |
| ConA + Collagen | **high** |
| Plasma-treated | **no** |

**Supplementary Table S1. Effects of various substrate-coating materials on the cell adhesion and growth**. The assessment of fungal cell adhesion was performed by counting hyphae left attached to the coverslip after changing the solution in 4 fields of view (20x 0.8 objective lens) on a bright-field microscope. **Very high** adhesion strength: more than 80 hyphae per field; **High**: between 40-80 hypha per field; **Medium**: between 10-40 hypha per field; **Low**: between 1-10 hypha per field; **No**: less than 3 hyphae per entire coverslip.

**Supplementary text**

**Effect of various treatments used during cell wall nanosurgery on hypha**

Several experimental manipulations used in our protocol could potentially have impact on the fungi cells. The prolonged incubation (more than 15 min) in hyperosmotic solutions, inducing plasmolysis, could have a negative impact on hypha. We assessed the fungi culture growth under medium (760 mOsm) and harsh (860 mOsm) hyperosmotic conditions, for 30 and 90 min, the typical and maximal duration of the entire hyperosmotic incubation during nanosurgery+patch-clamp experiment. As can be seen in Fig. S1a, 760 mOsm hyperosmotic conditions did not have significant effect on fungi growth at either time point examined, while harsh treatment induced significant reduction of fungi biomass yield after 30 min. During prolonged incubation adaptation to harsh treatment occurred, causing the absence of the effect of hyperosmotic conditions on the culture growth.

The effects of treatment by hyperosmotic solution were monitored in the separate set of hypha respiration measurements. Both hyperosmotic treatments, medium and harsh, had significant effect on fungi respiration (Fig. S1b), reducing it by 11% and by 30% respectively, while still allowing for the majority of respiration. Based on those results, we chose to use mild hyperosmotic treatment, 620 mOsm, as to minimize the negative influence of plasmolysis on fungi cells and insure that obtained protoplasts will be viable with intact plasma membrane.

We also assessed fungi cell respiration in a solution with sodium azide (NaN_3_) of different molarities, in order to find the concentration that would sufficiently inhibit respiration, to ensure that cell wall synthesis is slowed-down (Fig. S1c). Based on this curve, we chose to use 2 mM NaN_3_, a concentration slightly larger than the maximal tested 1.5 mM, at the plateau of maximal inhibition (83 ± 1 %). We also measured the long term effect (up to 4 h) on the respiration of NaN_3_ (5 mM) treated hyphae. As shown in Fig. S1d, the inhibition of respiration occurs quickly, it is already fully developed after 15 min and this initial very strong inhibition of respiration in high NaN_3_ concentration is maintained during the first 60 min of treatment. At all longer times of treatment with NaN_3_, fungi respiration recovers to 25-30% of initial value. This result is in accord with known hyphal cells ability to partially recover from respiration inhibition under various conditions^54^ and it shows that sodium azide is reliable and potent inhibitor of *Phycomyces* *blakesleeanus* respiration.


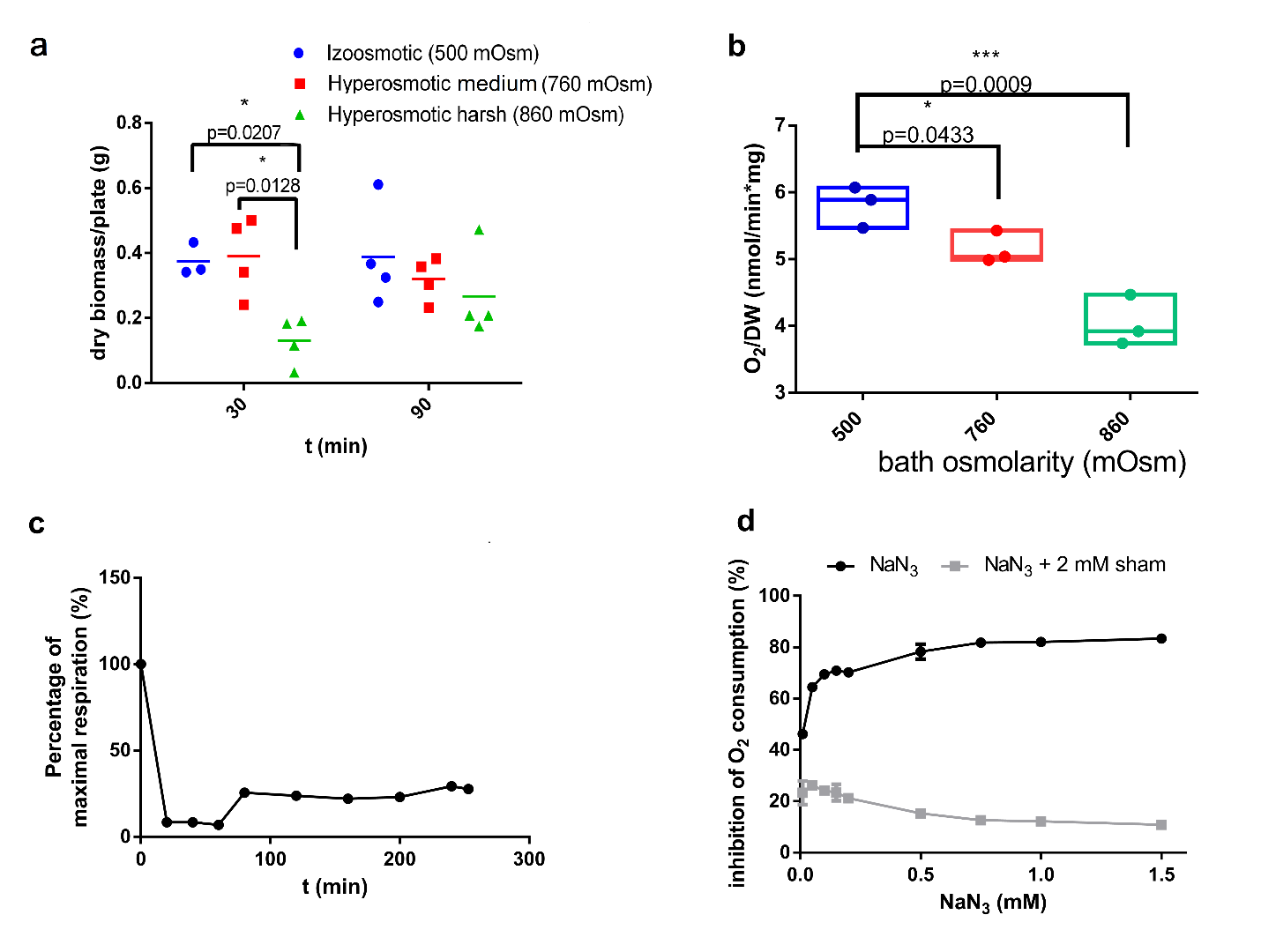

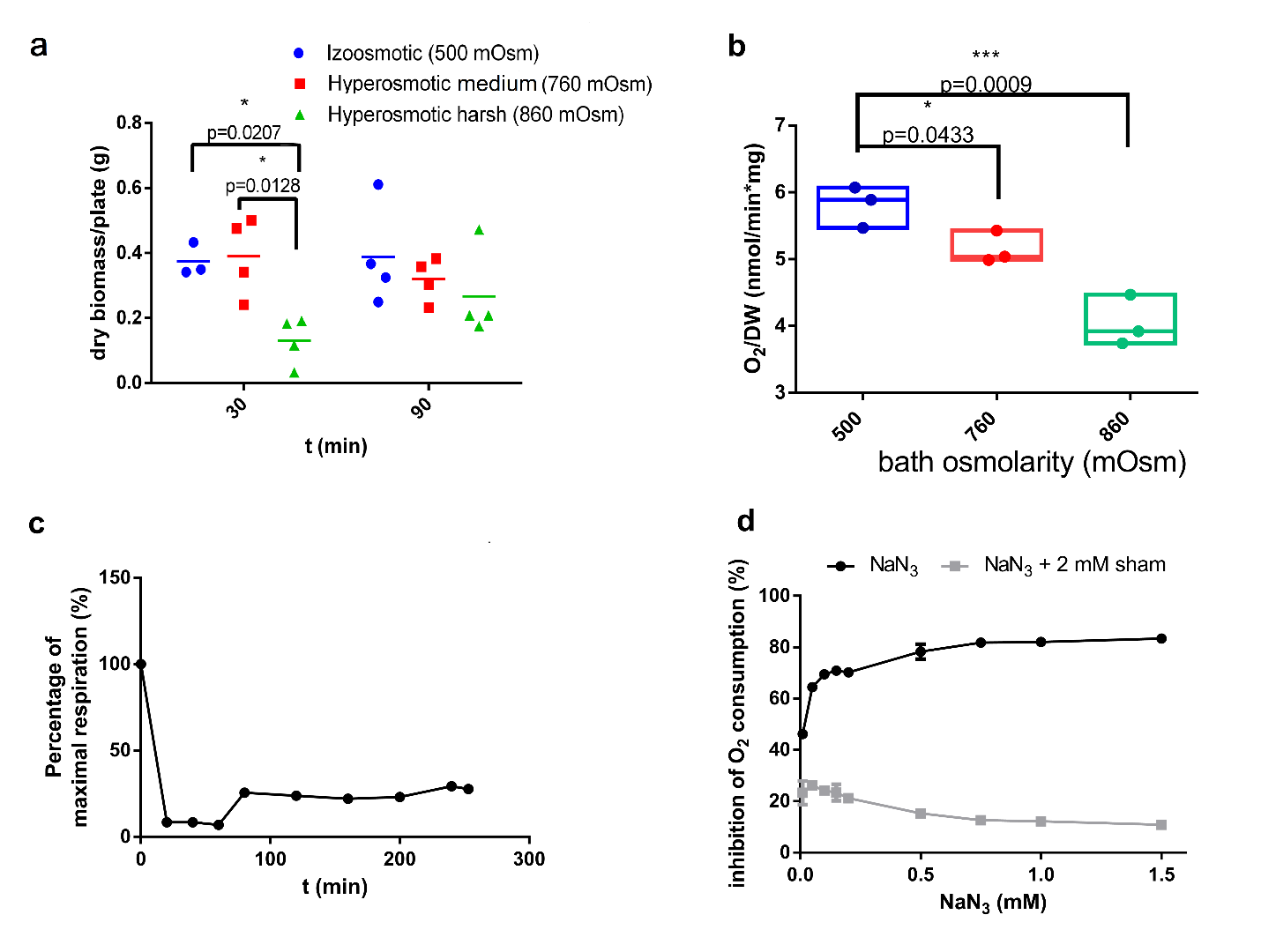

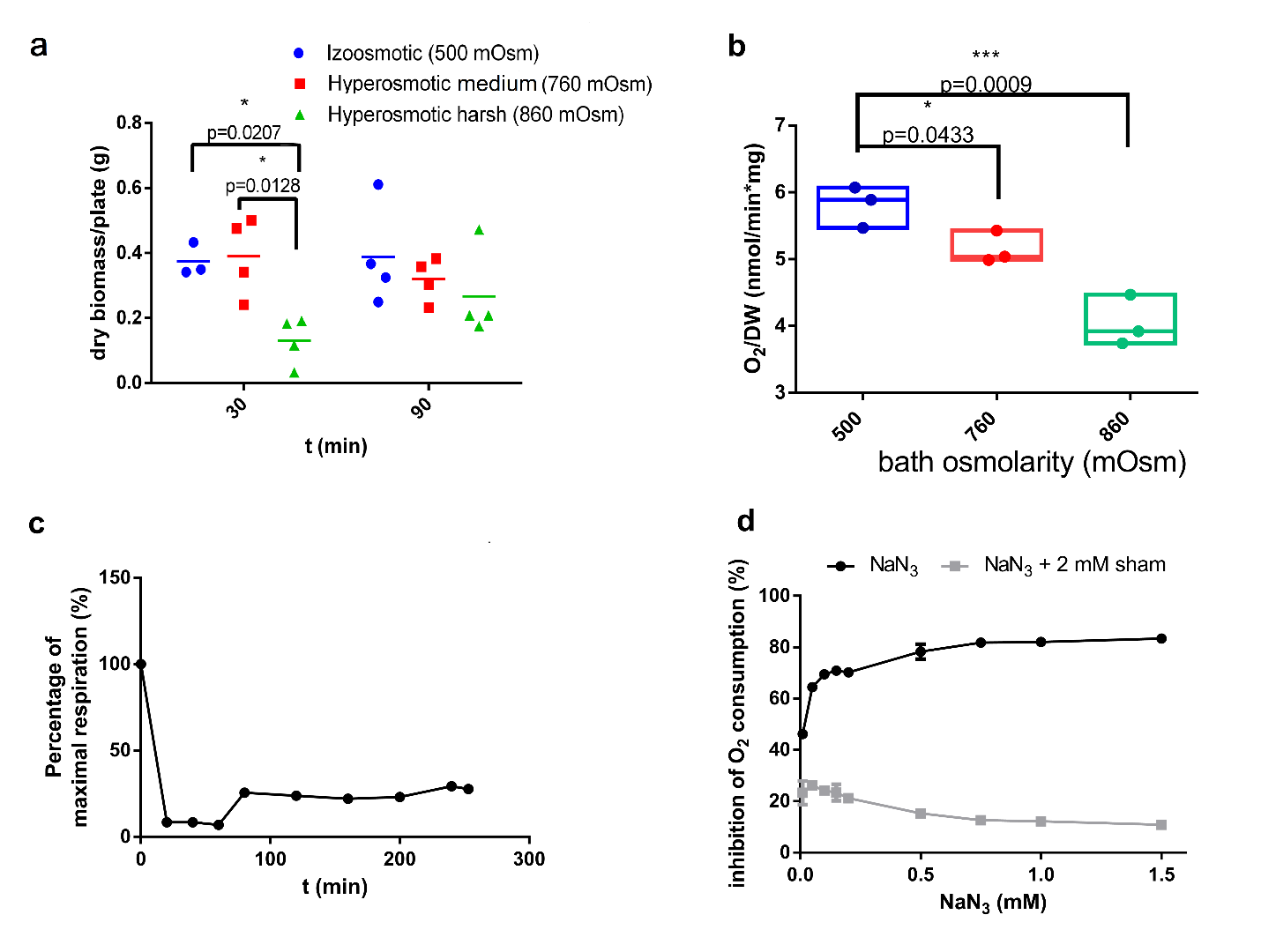


**a**

**b**

**c**

**d**

**Figure S1.** **Respiration and growth of *Phycomyces blakesleeanus* under conditions used for nanosurgery (hyperosmotic solution and respiration in sodium azide). a** Growth of petri dish culture, expressed as a yield of fungal mass, in solutions of increased osmolarity (after 30 and 90 min incubation). n=3-4. **b** Respiration of fungi culture after 90 min in solutions of increased osmolarity, expressed as oxygen consumption per minute, normalized to dry weight mass of culture. Box plot with line at median. n=3. **c** Dose dependent inhibition of respiration of fungi culture by NaN_3_, expressed as percent of O_2_ consumption of control that was inhibited by given concentration. n=3. Inhibition of respiration by SHAM (salycilhydroxamic acid) is given for comparison. 2 mM SHAM (inhibitor of alternative oxidase) was applied after NaN_3_ to verify the existence of at least minimal electron transport which enables viability of fungal hyphae. **d** Long-term inhibition of respiration by NaN_3_.

55. Stanić, M. *et al.* Effect of long-term cyanide exposure on cyanide-sensitive respiration and phosphate metabolism in the fungus phycomyces blakesleeanus. *Arch. Biol. Sci.* **66**, 847–857 (2014).
